# Psychosocial factors and patient and healthcare delays in large (class T3–T4) oral, oropharyngeal, and laryngeal carcinomas

**DOI:** 10.1186/s12885-024-12517-x

**Published:** 2024-06-25

**Authors:** Markus Atula, Timo Atula, Katri Aro, Heikki Irjala, Elina Halme, Anna Jouppila-Mättö, Petri Koivunen, Tommy Wilkman, Antti Mäkitie, Marko Elovainio, Laura Pulkki-Råback

**Affiliations:** 1 Department of Otorhinolaryngology-Head and Neck Surgery, University of Helsinki, Helsinki University Hospital, P.O.Box 263, Helsinki, HUS FI-00029 Finland; 2 Department of Otorhinolaryngology-Head and Neck Surgery, Turku University Hospital, University of Turku, Turku, Finland; 3 Department of Otorhinolaryngology-Head and Neck Surgery, Tampere University Hospital, University of Tampere, Tampere, Finland; 4 Department of Otorhinolaryngology-Head and Neck Surgery, Kuopio University Hospital, University of Eastern Finland, Kuopio, Finland; 5 Department of Otorhinolaryngology-Head and Neck Surgery, Oulu University Hospital, University of Oulu, Oulu, Finland; 6 PEDEGO Research Unit, University of Oulu, Oulu, Finland; 7 Medical Research Center Oulu, Oulu, Finland; 8 Department of Oral and Maxillofacial Surgery, University of Helsinki, Helsinki University Hospital, Helsinki, Finland; 9 Department of Clinical Sciences, Intervention and Technology, Division of Ear, Nose and Throat Diseases, Karolinska Institutet, Karolinska University Hospital, Stockholm, Sweden; 10 Research Program in Systems Oncology, Faculty of Medicine, University of Helsinki, Helsinki, Finland; 11 Research Program Unit, Faculty of Medicine, University of Helsinki, Helsinki, Finland; 12 Finnish Institute for Health and Welfare, Helsinki, Finland; 13 Department of Psychology and Logopedics, Faculty of Medicine, University of Helsinki, Helsinki, Finland

**Keywords:** Patient delay, Healthcare delay, Psychosocial factors, Socioeconomic status, Head and neck cancer, Cancer awareness

## Abstract

**Background:**

Psychosocial factors and socioeconomic status have been associated with incidence, survival, and quality of life among patients with head and neck cancer. We investigated the association between different psychosocial factors, socioeconomic status, and patient delays in T3–T4 oral, oropharyngeal, and laryngeal cancer.

**Patients and methods:**

We conducted a nationwide prospective questionnaire-based study (*n* = 203) over a 3-year period.

**Results:**

We found no association between psychosocial factors (depression, social isolation, loneliness, and cynical hostility) and patient delay. Depression was three times more common among head and neck cancer patients compared with the general Finnish population. Head and neck cancer patients had lower educational levels and employment status, and were more often current smokers and heavy drinkers.

**Conclusions:**

Although we found no association between patient delay and psychosocial factors, patients diagnosed with a large head and neck cancer appeared to have a lower socioeconomic status and higher risk for developing depression, which should be considered in clinical practice.

**Supplementary Information:**

The online version contains supplementary material available at 10.1186/s12885-024-12517-x.

## Background

Psychosocial factors and socioeconomic status (SES) have been associated with head and neck cancer (HNC) incidence, survival, and quality of life [1–5]. In many Western countries, absolute social inequalities in mortality are decreasing but relative inequalities are increasing [6–8]. Notably, the general health of various socioeconomic groups is improving, albeit at a different rate among specific sub-populations, thereby providing a greater benefit to people with a high SES. Specific to cancer, there is a clear difference in incidence and cancer mortality between people with high a SES versus a low SES, especially in cancers associated with tobacco and alcohol use and those related to chronic infections [9–11].

The social environment may impact individuals through exogenous exposures (viral, or occupational exposures) or behavioral exposures (tobacco smoking, or alcohol use). Smoking prevalence and alcohol consumption habits differ across SES groups [12–14]. For instance, a large meta-analysis published by Conway et al., consisting of 31 studies among 23 964 HNC patients and 31 954 controls, found that a low SES associated with an increased risk of developing HNC, with approximately one-third of that increased risk unexplained by differences in smoking and alcohol use and risk remaining elevated even among never smokers and nondrinkers [15]. Another meta-analysis by Conway et al. on oral cancer, consisting of 41 studies among 15 344 oral cancer patients and 33 852 controls, reported that individuals with an increased risk of developing oral cancer had a lower level of education, a low occupational status, and a low income [16]. Furthermore, according to two recent large studies by Weizman et al. and Bedir et al., consisting of 11 826 and 20 821 HNC patients, respectively, patients with a low SES exhibited a significantly lower overall survival compared with the most affluent patients [17, 18].

In addition, social relationships or a lack of them can carry a negative impact on individuals’ health [19]. Multiple pathways might plausibly explain how a lack of social relationships affects health [20]. First, psychosocial processes can affect malignancies by triggering stress responses in the autonomic nervous system and the hypothalamus–pituitary–adrenal axis [21], inducing a cascade of downstream processes that can affect tumor pathogenesis in multiple ways [22]. Second, a lack of social relationships is associated with behavioral factors (such as smoking and alcohol use), known risk factors for cancer [23]. Third, socially isolated individuals may experience a greater degree of mental health problems, such as depression, which has been associated with an increased overall cancer incidence [2], worse overall survival [3, 4], and a lower quality of life [5] in HNC.

Moreover, delays before initiating treatment lead to disease progression and a worse survival in HNC [24, 25]. Larger tumors often require multimodal treatment which is expensive and leads to poorer functional results. Thus, identifying factors influencing delays remains crucial. We previously examined patient and healthcare delays in large (class T3–T4) oral, oropharyngeal, and laryngeal carcinomas [26]. In this study, therefore, we investigated psychosocial and socioeconomic factors and their associations with delay in seeking medical care among patients with oral, oropharyngeal, and laryngeal carcinomas. We also examined how these factors differ between this patient cohort and the general Finnish population.

## Patients and methods

We conducted a nationwide questionnaire-based study covering all five university hospital districts in Finland. The patient population was previously described in detail [26]. Briefly, we included patients with a newly diagnosed T3–T4 oral, oropharyngeal, or laryngeal squamous cell carcinoma (SCC) treated between 1 September 2017 and 31 August 2020. In total, 528 patients met the inclusion criteria. We excluded patients incapable of understanding the questionnaire (*n* = 31), due to a language barrier (*n* = 5), or for other reasons (*n* = 7), or with an incapacity to participate due to an overall poor health condition (*n* = 19). Ten patients refused to participate, and 108 patients were not recruited because we were unable to deliver the questionnaire. Thus, the questionnaire was distributed to 348 patients, from whom 145 did not return it. In total, the final study cohort consisted of 203 patients. Oral cavity was the most common cancer site (*n* = 98; 48%), followed by oropharynx (*n* = 69; 34%) and larynx (*n* = 36; 18%). Most cancers were T4 (*n* = 116; 57%) and presented with lymph node metastases (*n* = 115; 57%).

Data were collected from patient questionnaires and patient hospital records. Patients received the questionnaire following a diagnosis but before the treatment initiation. If the questionnaire was not returned within a reasonable period of time, a reminder was sent via mail. The Research Ethics Board at the Hospital District of Helsinki and Uusimaa approved the study design (record number: 398/13/03/02/15) and an institution-specific permit to conduct this research was also received from each university hospital.

Patient delay was defined as the period between the patient’s reported symptom onset and the first contact with a healthcare provider. We also analyzed the following healthcare delays: primary healthcare delay (PHC), or the time period between the first contact with a healthcare provider and referral to a specialist care unit; and a specialist care (SC) delay, or the time period between the referral to a specialist care unit and the start of curative treatment.

The questionnaire included fixed, multiple-choice questions regarding education, employment, the household financial situation, and the patient-perceived health and functional capacity (Table 1). The questionnaire used in this study can be found in Supplementary File 1. To collect data on psychosocial factors, we used commonly used and validated questionnaires including the Social Support Questionnaire (SSQ), the Three-Item Loneliness Scale, Beck Depression Inventory (BDI), and the Cynical Distrust Scale (Table 2). Patients reported the time of onset of the first symptoms and the time of contact with a healthcare provider. Data collected from medical records included patient- and disease-related factors (Table 1).

**Table 1:** Median (Interquartile Range) delay in days and patient characteristics (*n* = 203 patients)

	Number [%]		Patient delay	*p* value	PHC^a^ delay	*p* value	SC delay^b^	*p* value
All patients								
Median delay			58 (94)		13 (38)		43 (30)	
Mean delay			102		43		51	
Range			0-943		0-825		12-244	
Age (in years)				0.726		0.520		0.234
< 40	5	[2.5]	61 (349)		7 (102.5)		26 (40.0)	
40-60	43	[21.2]	38 (75)		22 (40)		47 (24.5)	
> 60	155	[76.4]	59 (96)		12 (35)		42.5 (30.25)	
Sex				0.744		0.211		0.816
Male	142	[70.0]	57.5 (98)		12.5 (35.25)		43 (28.5)	
Female	61	[30.0]	59 (75.5)		17 (58.5)		43 (27)	
Education				0.035^e^		0.555		0.106
Primary school	85	[41.9]	62 (129.5)		13 (33)		48 (27)	
Secondary education^c^	76	[37.4]	58.5 (114)		13 (37.75)		43 (27.5)	
Post-secondary education^d^	36	[17.7]	31 (41.75)		23.5 (46.25)		35 (25.5)	
Data missing	6	[3.0]						
Employment				0.361		0.843		0.438
Currently employed or studying	34	[16.7]	34.5 (88.5)		22.5 (54.5)		41.5 (18.25)	
Unemployed	21	[10.3]	60 (189.5)		22 (37.5)		43 (33)	
Retired	134	[66.0]	59 (90.75)		12 (33.5)		44 (31)	
Other	7	[3.4]	30 (59)		28 (47)		67.5 (41.75)	
Data missing	7	[3.4]						
Marital status				0.423		0.108		0.102
Married or in a relationship	104	[51.2]	44.0 (108.75)		22.5 (38.75)		47.0 (27.0)	
Unmarried, divorced, or widowed	95	[46.8]	59.0 (70.0)		11.0 (32.0)		40.0 (28.0)	
Data missing	4	[2.0]						
Children living in the same household				0.060		0.098		0.511
No	180	[88.7]	59.0 (101.25)		12.5 (37.75)		43.0 (29.5)	
Yes	17	[8.4]	25.0 (71.0)		29.0 (47.0)		46.0 (22.0)	
Data missing	6	[3.0]						
Household size				0.194		0.085		0.235
One	93	[45.8]	59.0 (68.5)		11.0 (30.5)		41.0 (28.0)	
Two	90	[44.3]	49.0 (113.75)		15.5 (38.25)		47.0 (33.0)	
Three or more	16	[7.9]	28.0 (79.0)		30.0 (47.0)		46.5 (25.5)	
Data missing	4	[2.0]						
Household financial situation				0.481		0.496		0.710
Adequate funds	119	[58.6]	59 (82.0)		13 (37.0)		43 (28.75)	
Need to limit expenditures	59	[29.1]	42 (115.0)		13 (32.0)		43.5 (31.5)	
Receiving financial support	14	[6.9]	36.5 (118.25)		27 (54.0)		46 (29.0)	
Data missing	11	[5.4]						
Perceived general health				0.165		0.788		0.509
Excellent to very good	25	[12.3]	42 (88.0)		22 (38.0)		46 (17.0)	
Good	61	[30.0]	57 (133.0)		11 (30.0)		45 (29.75)	
Moderate to poor	53	[26.1]	78 (103.0)		12 (35.0)		42 (28.0)	
Data missing	64	[31.5]						
Perceived functional capacity (1-10)				0.758		0.875		0.181
Good to excellent (6-10)	118	[58.1]	51 (101.0)		13 (36.25)		42.5 (27.25)	
Poor to moderate (1-5)	36	[17.7]	79.5 (112.0)		17 (57.0)		47 (26.75)	
Data missing	49	[24.1]						

^a^*PHC* primary health care

^b^Specialist care delay, patients treated with a curative intent (*n* = 179)

^c^Senior high school

^d^University or university of applied sciences

^e^Primary school vs. post-secondary education, *p* = 0.033

**Table 2:** Median (Interquartile Range) delay in days and psychosocial factors (*n* = 203 patients)

	Number [%]		Patient delay	*p* value	PHC^a^ delay	*p* value	SC delay^b^	*p* value
Beck Depression Inventory				0.333		0.226		0.213
Normal (0-13)	139	[68.5]	46 (81.0)		16 (35.0)		42 (27.25)	
Mild (14-19)	26	[12.8]	50 (121.5)		10.5 (32.5)		56 (37.0)	
Moderate to severe (20 and over)	35	[17.2]	76 (104.0)		6 (48.0)		49 (25.5)	
Data missing	3	[1.5]						
Cynical Distrust Scale				0.464		0.607		0.694
Low (0-16)	122	[60.1]	54 (99.0)		13 (38.25)		43.5 (33.0)	
High (17-24)	43	[21.2]	61 (76.0)		12 (31.0)		48 (25.0)	
Data missing	38	[18.7]						
Social Support Questionnaire				0.435		0.483		0.121
Very high (9 and over)	38	[18.7]	57.5 (77.5)		10.5 (34.75)		48.5 (35.0)	
High (8 points)	27	[13.3]	53 (79.0)		23 (55.0)		44 (34.25)	
Moderate (5-7 points)	36	[17.7]	61 (90.25)		22 (38.0)		42 (32.0)	
Low (4 points)	77	[37.9]	43 (104.5)		11 (34.5)		40 (26.25)	
Very low (0-3 points)	22	[10.8]	80 (206.0)		14 (36.0)		51 (21.5)	
Data missing	3	[1.5]						
Loneliness				0.718		0.605		0.163
Never (3 points)	91	[44.8]	42 (83.0)		13 (33.0)		43 (23.75)	
Sometimes (4-5 points)	60	[29.6]	54 (67.0)		13.5 (51.25)		47 (33.0)	
Often (6-9 points)	38	[18.7]	60.5 (110.0)		17 (67.75)		48.5 (33.5)	
Data missing	14	[6.9]						
Do you have someone you can talk to?				0.969		0.760		0.322
No	9	[4.4]	30 (201.5)		18 (50.0)		38 (20.0)	
Yes	191	[94.1]	58 (86.0)		14 (38.0)		45 (28.25)	
Data missing	3	[1.5]						
Did someone tell you to seek medical attention?				0.003		0.904		0.716
No	80	[39.4]	31 (58.5)		12 (39.5)		44 (27.25)	
Yes	119	[58.6]	73 (128.0)		14 (38.0)		43 (30.0)	
Data missing	4	[2.0]						
Does someone close to you have cancer?				0.593		0.644		0.483
No	47	(23.2)	53 (177.0)		22 (33.0)		47 (31.5)	
Yes	153	(75.4)	58 (75.0)		13 (38.5)		43 (28.5)	
Data missing	3	(1.5)						
Were you aware of cancer risk factors?				0.274		0.159		0.556
No	25	[12.3]	31 (53.5)		4 (18.0)		39 (23.0)	
Very little	20	[9.9]	61 (55.5)		11.5 (29.0)		46.5 (21.0)	
To some degree	63	[31.0]	57 (107.0)		22 (44.0)		43 (31.0)	
Yes	91	[44.8]	61 (137.0)		16 (39.0)		47 (36.0)	
Data missing								
Did you suspect your symptoms might be caused by a cancer?				0.310		0.227		0.758
No	115	[56.7]	51 (77.0)		17 (37.0)		42 (25.0)	
Yes	81	[39.9]	61 (108.0)		11 (31.5)		46.5 (32.25)	
Data missing	7	[3.4]						

^a^*PHC* primary health care

^b^Specialist care delay, patients treated with a curative intent (*n* = 179)

To examine the degree to which the characteristics of our data were representative of the general Finnish population, we compared age- and sex-matched results from the present study to those obtained previously from the FinTerveys 2017 survey, consisting of 9288 randomly selected, geographically evenly distributed Finnish individuals aged 30 years or older (Table 3) [27]. Because FinTerveys was used only as a reference study, we did not perform any new analyses on these specific data.

**Table 3:** HNC patient cohort comparison to general Finnish population

	HNC cohort	General population	HNC cohort	General population	HNC cohort	General population	HNC cohort	General population	HNC cohort	General population
	40-49		50-59		60-69		70-79		80 +	
**Marital status**										
Men (n)	6		26		57		37		10	
Married or in a relationship	66.7	77.8	50.0	73.4	57.9	75.6	75.7	76.7	40.0	69.6
Unmarried, divorced, or widowed	33.3	22.2	50.0	26.6	42.1	24.4	24.3	23.3	60.0	30.4
Women (n)	3		7		19		13		16	
Married or in a relationship	33.3	76.1	57.1	72.2	31.6	65.3	38.5	53.9	12.5	23.7
Unmarried, divorced, or widowed	66.7	23.9	42.9	27.8	68.4	34.7	61.5	46.1	87.5	76.3
**Household size**										
Men (n)	6		26		57		37		10	
One	33.3	16.3	50.0	23.1	42.1	22.9	24.3	23.2	60.0	28.0
Two	33.3	21.8	38.5	46.8	52.6	69.1	73.0	72.1	40.0	68.8
Three or more	33.3	61.9	11.5	30.1	5.3	8.0	2.7	4.7		3.2
Women (n)	3		7		19		13		16	
One	33.3	12.2	42.8	21.2	63.2	32.2	61.5	44.3	87.5	73.1
Two		24.5	28.6	50.5	26.3	62.2	38.5	52.2	12.5	24.8
Three or more	66.7	63.3	28.6	28.3	10.5	5.6		3.5		2.1
**Education**										
Men (n)	6		26		57		35			10
Primary school	33.3	9.0	30.8	18.4	43.9	32.1	42.9	40.0	50.0	58.3
Secondary education	50.0	45.6	46.1	44.1	43.9	35.1	48.6	27.1	10.0	17.9
Post-secondary education	16.7	45.4	23.1	37.5	12.2	32.8	8.5	32.9	40.0	23.9
Women (n)	3		7		19		13		16	
Primary school	33.3	6.4	28.5	9.3	42.1	28.1	61.5	50.7	68.8	63.8
Secondary education	33.3	27.3	43.0	31.4	42.1	31.4	15.4	23.3	6.2	20.7
Post-secondary education	33.3	66.3	28.5	59.3	15.8	40.5	23.1	26.0	25.0	15.4
**Employment**										
Men (n)	6		26		57					
Currently employed/studying	50.0	88.1	50.0	75.0	10.5	24.3				
Unemployed	50.0	7.6	28.6	11.6	5.3	5.7				
Retired		3.5	15.4	10.3	78.9	68.2				
Other		0.8	3.8	3.1		1.8				
Women (n)	3		7		19					
Currently employed/studying	33.3	86.8	71.4	79.5	10.5	21.0				
Unemployed	66.7	6.4	28.6	9.6	15.8	5.3				
Retired		1.4		5.0	73.7	71.1				
Other		5.4		5.9		2.6				
**Household financial situation**										
Men (n)	6		26		55		35		9	
Adequate funds	33.3	62.4	42.3	61.3	58.2	68.4	80.0	66.9	77.8	64.7
Inadequate funds	66.7	37.6	57.7	38.7	47.8	31.6	20.0	33.1	21.2	35.3
Women (n)	3		7		17		13		16	
Adequate funds		56.3	42.9	62.7	52.9	63.3	69.2	56.7	81.3	59.5
Inadequate funds	100	43.7	57.1	37.3	47.1	36.7	30.8	43.3	18.7	40.5
**Current smokers (prior to diagnosis)**										
Men (n)	6		27		58		38		10	
No	16.7	70.2	33.3	72.2	37.9	78.1	50.0	89.1	90.0	98.4
Yes	83.3	29.8	66.7	27.8	62.1	21.9	50.5	10.9	10.0	1.6
Women (n)	3		7		19		14		14	
No	66.7	80.7	71.4	81.6	42.1	85.3	57.1	94.6	92.9	98.4
Yes	33.3	19.3	28.6	18.4	57.9	14.7	42.9	5.4	7.1	1.6
**Alcohol abstinence**										
Men (n)	6		26		54		35		8	
No alcohol use	16.7	11.0	26.9	13.3	29.6	17.2	34.3	20.3	25.0	40.2
Alcohol use	83.3	89.0	73.1	76.7	70.4	82.8	65.7	79.7	75.0	59.8
Women (n)	3		7		17		14		13	
No alcohol use	66.6	10.8	42.9	12.7	52.9	22.4	50.0	35.7	76.9	54.9
Alcohol use	33.3	89.2	57.1	87.3	47.1	77.6	50.0	64.3	23.1	45.1
**How often do you consume at least six units of alcohol?**										
Men (n)	6		27		51		37		9	
Never	16.7	16.1	29.6	21.7	31.4	28.9	37.9	47.5	33.3	73.7
Less than once a month	33.3	43.8	14.8	40.3	37.3	39.3	35.1	37.4	55.6	20.1
Once a month	50.0	25.1	33.3	20.7	9.8	18.0	13.5	9.4	11.1	3.2
Weekly		15.0	22.2	17.3	21.5	13.8	13.5	5.7		3.0
Women (n)	3		6		17		13		14	
Never	66.7	32.4	50.0	43.5	52.9	64.7	53.8	82.5	78.6	97.3
Less than once a month	33.3	57.5	16.7	42.5	35.3	28.2	46.2	14.7	21.4	2.1
Once a month		5.8		8.8	11.8	5.1		2.1		0.6
Weekly		4.3	33.3	5.3		2.0		0.8		
**Becks Depression Inventory**	30 - 64		65 +							
Men (n)	63		77							
Normal	73	91	75	93						
Mild to severe	27	9	25	7						
Women (n)	23		37							
Normal	48	87	65	89						
Mild to severe	52	13	35	11						
**It's better not to trust other people**										
Men (n)	6		22		47		32		5	
True or mostly true	16.7	21.7	27.3	22.4	40.4	27.2	37.5	34.4	60.0	38.9
False or mostly false	83.3	78.3	82.7	77.6	59.6	72.8	62.5	65.6	40.0	61.1
Women (n)	2		7		15		12		12	
True or mostly true	100	17.1	14.3	17.2	20.0	26.5		32.1	41.7	34.0
False or mostly false		82.9	85.7	82.8	80.0	73.5	100	67.9	58.3	66.0
**Average functional capacity (0-10)**										
Men (n)	4		22		43					
Mean score	7.3	8.3	7.2	7.5	7.0	6.8				
Women (n)	3		5		16					
Mean score	5.7	8.5	6.8	7.8	6.8	6.9				

The associations for patient and psychosocial characteristics with patient, PHC, and SC delays were analyzed using the Mann–Whitney *U* test and the Kruskal–Wallis test applying the Dunn–Bonferroni correction for pairwise comparisons. Statistical analyses were performed using IBM’s SPSS Statistics version 26 for Windows (IBM Corp., Armonk, NY, USA). We considered *p* < 0.05 as statistically significant.

## Results

In this patient series, the median patient delay was 58 days, with 70% of patients seeking medical care within three months, a finding reported earlier [26]. Patient characteristics and delays are summarized in Table 1. In our previous study, we reported the following findings: age, sex, education, and employment [26]. These previous and all other patient-related variables examined in the present study revealed that only education level significantly associated with patient delay. Psychosocial factors did not statistically associate with patient delay (Table 2).

The median patient delay was significantly longer among those who were prompted by others to seek medical care (73 days) compared with those who were not (31 days). The other psychosocial characteristics were not associated with delays, although we observed a tendency, whereby patients who exceeded the cutoff for moderate depression and patients who reported having low social support had longer delays than patients without these characteristics. The patient cohort comparison with the general Finnish population appears in Table 3.

## Discussion

In the current series, we found no association between psychosocial factors and patient delay. Compared to the general Finnish population, patients with large HNCs reported more depressive symptoms, were socially more isolated, and had a lower SES (Table 3).

Multiple psychosocial and socioeconomic factors appear to affect overall survival in HNC [3, 4, 17, 18]. Furthermore, delay before the initiation of curative treatment leads to worse overall survival in HNC [25, 28, 29]. In our previous study, we reported that the median patient delay for large oral, oropharyngeal, and laryngeal cancer was about two months [26], compared with a median of an approximately one-month delay in our two previous studies that also included early-stage diseases and all HNC sites [30, 31].

Depressive symptoms (BDI > 13) were present among 30% of HNC patients (13% mild; 17% moderate to severe symptoms) at the time of diagnosis, which agrees with the literature. According to a review of depression among HNC patients (*n* = 52 studies), depression rates were particularly high at diagnosis (13–40%, *n* = 21 studies), during treatment (25–52%, *n* = 7 studies), and at six-month follow-up (11–45%, *n* = 11 studies) [32]. Three recent studies consisting of 71 541, 55 069, and 3466 HNC patients, respectively, found that the prevalence of a major depressive disorder was 9.3%, 11.5%, and 18.5% [3, 4, 33]. Furthermore, two of these large studies showed that patients diagnosed with depression prior to an HNC diagnosis had a worse overall survival [3, 4].

We found no association between patient delay and depressive symptoms, a finding similar to a study by Rozniatowski et al. [34]. In our study, we could not study depressive symptoms prior to a cancer diagnosis, possibly impacting our result. Compared with the general Finnish population, depressive symptoms were more common among HNC patients (Table 3) [27]. We observed no association between cynical hostility and patient delay, and we found no other studies examining this issue. Compared with the general population, distrust was more common among male HNC patients [27]. It appears that distrust of authorities does not delay patients’ health-seeking behavior, although further research is needed.

A significantly larger portion of our study population was unmarried, divorced, or widowed (59%) compared with the general Finnish population (29%) aged 30 and older [27]. Similarly, more people were living alone (59% vs. 25%) and similar proportions of people were living in households of two (44% vs. 44%), but far fewer lived in households of three or more people (8% vs. 31%) [27]. We also found no association between delays and marital status or household size, although we observed a trend (*p* = 0.06) between shorter patient delay among patients with children living in the same household. Many other studies reported a similar findings [35–38], but not all [34]. In a study by Rozniatowski et al., patients presenting with a large primary tumor (T3–T4) were more often single, separated, divorced, or widowed [34]. Furthermore, patients’ perceptions regarding their social connections and support as well as feelings of loneliness did not affect patient delay in our series.

In addition, patients told to seek medical attention by someone else had significantly longer median patient delays (73 vs. 31 days) in our patient cohort. Initially, this sounds counterintuitive, but might be because patients who initially delayed seeking medical care were eventually told to do so by someone else. In a study by Rozniatowski et al., advice from a partner served as an important motivating factor for seeking medical attention [34].

In our previous study based on the same patient series, we found that patient education, but not employment, affected patient delay, a finding that remained significant in our multivariable model. However, the literature on this matter remains inconclusive [35, 38, 39]. The majority of our study population (59%) felt that their household financial situation was good (adequate funds), which mirrors that among the Finnish population of the same age (62%) [27]. We also found no association between perceived household income and patient delay, a finding similar to some studies [36, 38], but not all [35, 39].

In multiple studies, socioeconomic deprivation or low a SES associated with longer SC delay [24, 40, 41], but we observed no association between socioeconomic factors and PHC or SC delays. One contributing factor might be spatial access to treatment. For instance, in a Canadian study, the most deprived patients had the longest travel times to HNC treatment centers [42]. In Finland, governmental authorities regulate the management of HNC, which is entirely organized through the public healthcare system. Patients are treated in one of five university hospitals in accordance with national treatment guidelines. Patients can freely seek medical care from the public or private sectors, all of which can similarly refer patients to SC. If the patient chooses to contact the public healthcare system, it is almost entirely funded by the patients’ municipality. In Finland, there is a total patient expenditure limit of 683€ per year including all healthcare services, after which all healthcare services are entirely free for the patient. Thus, private health insurance is not widely used in Finland, possibly further explaining the differences in our study compared with some other studies, where private insurance, and, therefore, patients’ economic capabilities, play a larger role in healthcare-seeking behavior.

In our previous study, we described an association between longer patient delay among patients who reported current or former heavy alcohol use, but observed no association between patient delay and smoking status [26]. The literature on these issues remains inconclusive [30, 31, 38, 43–46]. Our current study revealed that compared with the general Finnish population there were more current smokers and heavy drinkers among patients with HNCs, as we expected, since these are well-known risk factors for developing HNC (Table 3). In addition, a lower SES has been associated with a higher smoking prevalence, [12] but contrary to tobacco smoking, alcohol consumption is more prevalent among high SES groups [13] and the prevalence of heavy episodic drinking (HED) is more common among high SES groups [14]. However, in Finland, this socioeconomic gradient has not been observed among men, and among women HED is more prevalent among the low SES group [47]. Still, the adverse health effects of alcohol consumption impact the low SES groups [13, 48, 49], including HNC [1].

In our series, most patients (76%) were aware of cancer risk factors at least to some degree and had a close relative with a history of cancer (75%). Many (40%) suspected that their symptoms might be caused by cancer, which is substantially higher than in an English study on oral and oropharyngeal cancer (13%) [50]. This difference might result from our inclusion of patients only with large tumors. That said, we observed no association between these factors and patient delay. Many studies on HNC have found an association between patients’ knowledge of cancer symptoms, their false interpretation of symptoms as benign, and longer patient delay [35, 36, 39, 45]. A large English study on cancer symptom awareness (including HNC symptoms) in the general population among nearly 50 000 people found that a lower symptom awareness significantly associated with being single or unemployed or having a lower SES [51].

One major limitation to our study was the large number of patients we were unable to recruit participate, possibly biasing our findings. We minimized the recall bias by double-checking the reported delay time points from hospital records to see if they matched. In addition, due to our study setting we were unable to determine patients’ psychological status prior to HNC diagnosis possibly impacting our results.

In our series, a comparison with the general population revealed that patients with a large primary tumor in the oral cavity, oropharynx, and larynx were more socially isolated, had a lower educational level and employment status, were current smokers and heavy drinkers, expressed more depressive symptoms, and their perceived functional capacity was lower (Table 3) [27]. This should be considered in the planning of public symptom awareness campaigns. A tailored approach targeting these HNC risk groups might be more effective, possibly resulting in a shorter patient delay. Although we found no association between patient delay and psychosocial factors, patients diagnosed with a large HNC seemed to have a lower SES and at a higher risk for developing depression, which should be considered in clinical practice.

### Supplementary Information

Supplementary Material 1.

## Data Availability

The data that support the findings of this study are available from the corresponding author, MA, upon a reasonable request.

## Abbreviations

BDI: Beck Depression Inventory
HED: Heavy episodic drinking
HNC: Head and neck cancer
PHC: Primary healthcare
SC: Specialist care
SCC: Squamous cell carcinoma
SES: Socioeconomic status
SSQ: Social Support Questionnaire
