# Supplementary material for: Psychosocial factors and patient and healthcare delays in large (class T3–T4) oral, oropharyngeal, and laryngeal carcinomas
Source: BMC Cancer. 2024 Jun 25;24:760. doi: 10.1186/s12885-024-12517-x (PMC11197283; doi:10.1186/s12885-024-12517-x)
Supplement: Supplementary file 1 — Supplementary Material 1. [file 12885_2024_12517_MOESM1_ESM.pdf]

Hospital ___________________

Patient number _____________

Patient questionnaire

Name:_______________________________________

We kindly ask you to answer to all the questions. Choose the most accurate option in your opinion or write an answer to the appointed line. If you can't recall everything accurately, answer according to your best approximation or memory of the events. Your answers to this questionnaire are confidential, so we kindly ask you to answer truthfully.

1. What signs or symptoms did you have before you contacted to a health-care provider? Choose one or multiple options.

☐ Pain

☐ A neck lump

☐ Difficulties swallowing

☐ Hoarseness

☐ Difficulties breathing

☐ Weight loss

☐ Ulcer or another change inside your mouth, throat, or nose

☐ Bleeding

☐ Facial swelling

☐ Facial nerve palsy

☐ Problems with eyesight

☐ An incidental finding regarding another investigation

☐ Other, what?

____

2. What was the most important initial symptom? Choose one.

☐ Pain

☐ A neck lump

☐ Difficulties swallowing

☐ Hoarseness

☐ Difficulties breathing

☐ Weight loss

☐ Ulcer of another change in mouth, throat or nose

☐ Bleeding

☐ Facial swelling

☐ Facial nerve palsy

☐ Problems with eyesight

☐ No symptoms

☐ Other, what?

____

3. Did you have some other reasons to seek medical care?

____

4. Did you experience pain?

☐ Painful, I needed to take painkillers regularly.

☐ Mildly painful, I needed to take painkillers occasionally.

☐ I was painless.

5. Did you suspect you might have cancer?

☐ Yes

☐ No

6. What is your marriage status?

☐ Unmarried

☐ Married or in a relationship

☐ Divorced or separated

☐ Widow

7. Do you have children living in your household?

☐ No

☐ Yes. How many? _______________

8. Do you have a person, with whom you can talk about things that are troubling you?

☐ Yes

☐ No

9. Did someone close to you urge you to seek medical attention?

☐ Yes

☐ No

10. Do you know closely someone who has had cancer?

☐ Yes

☐ No

11. Were you aware of the risk factors of head and neck cancer (tobacco smoking and alcohol drinking)?

☐ Yes

☐ To some extent

☐ Very little

☐ No

12. When did you first notice a sign or a symptom (date/month/year; as accurately as you can recall). Your most accurate estimation or recollection of the date is enough.

____

13. When did you seek medical attention (for example booked an appointment)?

____

14. When did you have your first appointment to a medical doctor regarding your symptoms (date/month/year)?

____

15. What health-care provider did you contact initially?

☐ Healthcare center, occupational healthcare or another medical doctor (excluding otorhinolaryngologists)

☐ Private otorhinolaryngologist

☐ A dentist

☐ Hospital emergency

16. What is your own impression on the health-care providers expression whether your condition was initially regarded to be most likely benign or malignant?

☐ Benign

☐ Malignant

☐ No impression

17. Were your signs/symptoms initially treated as an infection?

☐ Yes

☐ No

18. Were your signs/symptoms treated with antibiotics?

☐ No

☐ Yes, how many courses of antibiotics? ________________

19. Were your signs/symptoms treated as asthma?

☐ Yes

☐ No

20. What was agreed on at the initial visit?

☐ A referral to specialist care unit was made

☐ I received treatment and/or diagnostic examinations and a control visit was scheduled

☐ I received treatment and/or diagnostic examinations but NO control visit

☐ No treatment and/or diagnostic examinations and no control visit

21. How many visits to a medical doctor did you have before the referral to the specialist care unit was made?

____

22. When was the last time you visited a dentist (your most accurate recollection)?

____

23. Do you smoke cigarettes?

☐ I have never smoked

☐ I have quit smoking in ________(year). When I smoked, I smoked _____cigarettes (or cigars) per day on average.

☐ Yes, ___________ cigarettes (or cigars) per day on average.

24. If you previously chose **the second** or **the third** option, estimate for how long have you smoked (currently or previously)?

I have used tobacco products for _________ years.

25. How many drinks of alcohol do you consume in a week on average? One drink of alcohol is a can/small bottle of beer, cider or long drink (0,33 litres), 12 cl of wine or 4 cl of spirits.

_________ drinks.

26. How many times have you consumed over 5 drinks of alcohol on one occasion in the last 30 days?

_____________ times.

27. Have you had a time in your life when you have used alcohol excessively (regularly over 15 drinks of alcohol for men or 8 drinks of alcohol for women)?

☐ Yes

☐ No

28. Choose your highest education

☐ Primary school

☐ Vocational school

- ☐ Secondary school (high school)
- ☐ University of applied sciences
- ☐ University
- ☐ Other, what? ________________________

29. How would you describe your general health condition?
    - ☐ Excellent
    - ☐ Very good
    - ☐ Good
    - ☐ Moderate
    - ☐ Bad

30. How would you describe your functional capacity (how well are you managing your daily housework)? *Give us your estimation of your functional capacity in a scale of 1-10, 10 being the highest possible value.*

    ____

31. Your employment status
    - ☐ Working
    - ☐ Unemployed
    - ☐ Studying
    - ☐ Retired
    - ☐ Other

32. Occupation

    ____

33. How would you describe the financial situation of your household?
    - ☐ We have enough income to fill our daily needs and we can save money for future.
    - ☐ We need to monitor our spending.
    - ☐ We get supplementary benefits from the government.

34. Free comment regarding the function of healthcare system.

____

____

Psychosocial factors in seeking medical care

1. **Evaluate your possibilities to get assistance or comfort when you need it.**

   You can choose one or multiple options.

	**Spouse, Partner**	**Some other close relative**	**A close friend**	**A close co-worker**	**A close neighbour**	**Some other close acquaintance**	**No one**
Whom can you really count on to help you feel more relaxed when you are under pressure or tense?	1	2	3	4	5	6	7
Whom can you really count on to care about you, regardless of what is happening to you?	1	2	3	4	5	6	7
Whom can you really count on to help you feel better when you are feeling generally down-in-the-dumps?	1	2	3	4	5	6	7
Whom can you really count on to get hands-on help when you need it?	1	2	3	4	5	6	7

2. **Answer to the next questions based on how often you feel the certain way.**

	Hardly ever	Some of the time	Often
How often do you feel that you lack companionship?	1	2	3
How often do you feel left out?	1	2	3
How often do you feel isolated from others?	1	2	3

3. **Pearson / Hoegrefen hold the copyright for Beck Depression Inventory (BDI) and therefore it can not be presented here. The questionnaire can be found here:**

   https://www.hogrefe.fi/tuote?product_id=531

4. **Social relationships**

   Read the following claims and circle what you feel to hold most accurately true.

	Is completely true	Is pretty much true	Not exactly true	Not true at all
I think most people would lie to get ahead	1	2	3	4
Most people are honest chiefly through fear of being caught	1	2	3	4
Most people will use somewhat unfair means to gain profit or an advantage rather than lose it	1	2	3	4
I commonly wonder what hidden reason another person may have for doing something nice to me	1	2	3	4
No one cares much what happens to you	1	2	3	4
It is safer to trust nobody	1	2	3	4
Most people make friends because friends are likely to be useful to them	1	2	3	4
Most people inwardly dislike putting themselves out to help other people	1	2	3	4
